# Current treatment and surveillance modalities are not sufficient for advanced stage III colon cancer: Result from a multicenter cohort analysis

**DOI:** 10.1002/cam4.4417

**Published:** 2021-11-16

**Authors:** Juan Li, Yumo Xie, Ziying Huang, Dingcheng Shen, Zhuokai Zhuang, Mingxuan Zhu, Yaoyi Huang, Rongzhao He, Xiaolin Wang, Meijin Huang, Yanxin Luo, Huichuan Yu

**Affiliations:** ^1^ Department of Colorectal Surgery The Sixth Affiliated Hospital Sun Yat‐sen University Guangzhou Guangdong China; ^2^ Guangdong Provincial Key Laboratory of Colorectal and Pelvic Floor Disease Guangdong Institute of Gastroenterology The Sixth Affiliated Hospital Sun Yat‐sen University Guangzhou Guangdong China; ^3^ Zhongshan School of Medicine Sun Yat‐sen University Guangzhou Guangdong China

**Keywords:** advanced stage, colorectal cancer, management, SEER, survival, treatment, tumor staging

## Abstract

**Objective:**

We conducted this multicenter cohort study to evaluate the current tumor‐node‐metastasis staging system and treatment modality by analyzing the survival outcomes of patient groups with stage III and IV colon cancer.

**Patients and Methods:**

Stage III and IV colon cancer patients from the Surveillance, Epidemiology, and End Results (SEER) database (SEER cohort) and prospectively maintained Sun Yat‐sen University (SYSU) cohort were included in this study. Kaplan‐Meier method was used to estimate the cumulative rate of overall survival (OS) between patient groups, and the inverse probability weighting method was used to calculated age and sex‐adjusted survival curves. The Cox regression model was used to identify the risk factors for OS.

**Results:**

A total of 17,911 and 1135 stage III–IV cases were included in the SEER and SYSU cohorts, respectively. Among them, 1448 and 124 resectable stage IV cases underwent curative‐intent treatment in the SEER and SYSU cohorts, respectively. The T4N2b group showed a significantly worse survival outcome compared with the M1a subset receiving curative‐intent treatment (HR, 1.46; *p *< 0.001). This finding was validated in the SYSU cohort, in which the T4N2 group had a worse outcome than that of the M1 group receiving curative‐intent treatment (HR, 2.44; *p *< 0.001). These findings were confirmed in the adjusted survival analysis. In the multivariate analysis, the right‐side tumor, poor‐undifferentiated tumor, and age over 60 years were identified as independent risk factors for T4N2b patients. Based on this multivariate model, the high‐risk T4N2b subgroup had a worse survival outcome compared with resectable M1b patients (HR, 1.24; *p *= 0.03).

**Conclusion:**

By comparing stage III with stage IV colon cancer patients, we identified a subgroup of stage III patients at a higher risk of death than more advanced stages, implying that current cancer care modalities are not sufficient for these high‐risk substages.

## Graphical abstract

By analyzing the Surveillance, Epidemiology, and End Results database and in‐house cohorts, several stage III subgroups were identified in a high risk of death, especially the T4N2b group that had significantly worse overall survival than that of the M1a subset. When further categorized by risk factors, the survival of the high‐risk T4N2b patients was worse than that of the M1b subset.

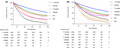

## 1 INTRODUCTION

Colorectal cancer (CRC) is the third common malignancy and the second leading cause of cancer death worldwide.^1^, ^2^, ^3^ It was estimated that CRC comprises 10% (1.9 million) of global new cancer cases and 9.4% (0.9 million) of cancer death in 2020.^4^ To predict the outcomes and guide the clinical decisions, multiple tumor staging systems have been employed to select patients for optimal care in CRC.^5^, ^6^, ^7^ Among them, the American Joint Committee on Cancer (AJCC) tumor‐node‐metastasis (TNM) classification system is the most prevalent system for CRC staging.

TNM staging has been recognized as a system that could stratify patients by risk of tumor recurrence, metastasis, and death. The current TNM staging system has been widely used to guide clinical decisions and surveillance strategies and serves as the basic selection criteria and the reference for clinical trials. The majority of CRC clinical guidelines draft their recommendations of cancer care based on the TNM categories.^8^, ^9^, ^10^, ^11^ In particular, in colon cancer, targeted therapy, immunotherapy, and more frequent surveillance are currently only recommended for stage IV disease regarding its poorer survival outcome and potential survival benefit. Considering the additional toxicities, cost‐effectiveness, and lack of established clinical evidence, these treatments are not extended to other stages currently. However, heterogeneity with treatment response and survival outcomes does exist within each TNM stage in CRC. Some subgroups at earlier stages demonstrated worse survival outcomes than advanced stage and even metastatic stage.^12^, ^13^

Therefore, the primary objective of this study is to validate the current TNM staging system for colon cancer, and the secondary objective is to identify the substages that had unexpectedly worse survival outcomes than more advanced stages by analyzing the survival outcomes of different subcategories among stage III and IV patients in the Surveillance, Epidemiology, and End Results (SEER) database and our in‐house cohort with convinced populations and robust power. We speculated that current cancer care modalities are not sufficient for these high‐risk substages with poor clinical outcomes.

## 2 MATERIALS AND METHODS

### 2.1 Patients

**FIGURE 1:**
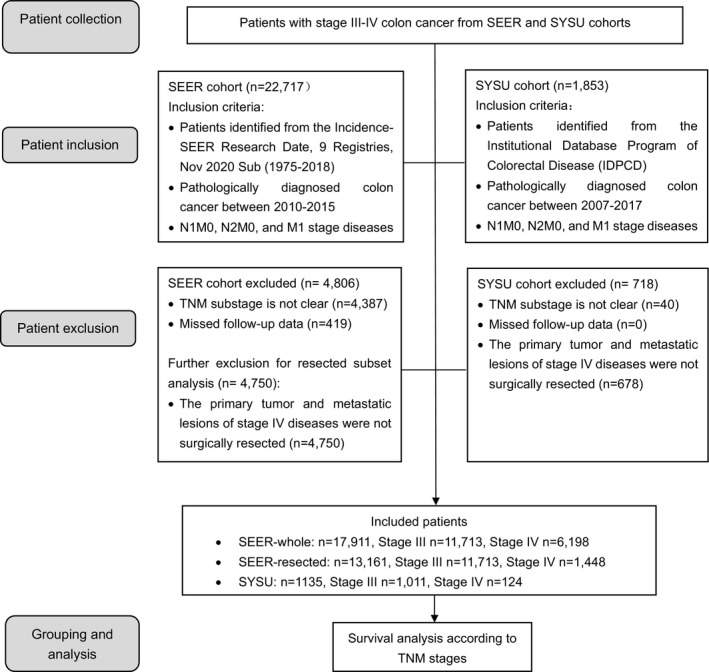
Flow diagram for patient disposition in the study

The inclusion criteria of the total cohort from the SEER database (Incidence‐SEER Research Date, 9 Registries, Nov 2020 Sub (1975–2018))^14^ were stage III patients with resected primary tumors and those with IV colon cancer diagnosed from 2010–2015. The excluding criteria were without available follow‐up data. The flow chart is shown below (Figure 1). The curative‐intent cohort within the total cohort of the SEER database was stage III patients and stage IV patients with performed metastasectomy. The primary endpoint was overall survival (OS) that was defined as the time from the diagnosis to the death from any cause or the last follow‐up. The final SEER total cohort including in the analysis has 17,911 cases, and the curative‐intent group consists of 13,161 patients. The seventh edition of the TNM classification system has been applied from 2010–2017^15^ with a more detailed subgroup classification of T stage, N stage, and M stage than the sixth edition.^16^ All staging data were pathologically confirmed and recorded according to the 7^th^ edition of the AJCC TNM staging system.

A total of 1135 histopathologically confirmed stage III and IV colon cancer patients receiving curative‐intent treatment at the Sixth Affiliated Hospital of Sun Yat‐sen University (SYSU cohort) from January 2007 to December 2017 were included in this study. The clinical and prognostic data of the SYSU cohort were collected from the prospectively maintained Institutional Database Program of Colorectal Disease (IDPCD) as previously described.^17^, ^18^ The 7th edition AJCC staging system was applied in the SYSU cohort patients diagnosed between 2007–2009 to achieve a unified pathological staging for analysis. Patients not achieving R0 resection for primary and metastatic lesions with gross or microscopic tumor residues were excluded in this cohort. The survival duration was defined as the time between surgery and the last date that survival information was collected or the day of confirmed death.

### 2.2 Treatments

Patients in the SYSU cohort were treated and followed up according to NCCN guidelines‐based protocols in our institute.^19^, ^20^, ^21^, ^22^ For stage IV cases, the treatments of metastases included surgical resection, radiofrequency ablation, and microwave ablation, which had been preoperatively evaluated and conducted by the multi‐disciplinary team. Over 70% of patients had chemotherapies. The 5‐FU based chemotherapy regimens, including FOLFOX XELOX and Capecitabine/deGramont, were applied for stage III and IV patients. The targeted therapies, including bevacizumab and cetuximab, were only used in stage IV patients. The therapeutic modalities for stage III and IV after recurrence were the same, consisting of surgical resection, ablation, chemotherapies, and targeted therapies.

### 2.3 Statistical analysis

Individual variables were compared by using the Mann‐Whitney *U* test, Student's *t*‐test, χ^2^ test, or Fisher's exact test according to their types and distributions. Overall survival was the primary endpoint. The cumulative rate of OS was estimated by the Kaplan‐Meier method. Differences were evaluated using the log‐rank test. The baseline variables, including age, gender, race, the primary site of tumor, tumor histology/behavior, and the year of diagnosis, were weighted by the inverse probability weighting (IPW) method,^23^ and the R package “Survival” was used to plot the IPW‐adjusted survival curves. Risk factors for OS were analyzed by the Cox proportion hazards regression model. The variables with a *p* value <0.10 in univariate analysis were further evaluated in a multivariate analysis using the backward stepwise elimination method with a removal cutoff of *p* = 0.10. All tests were 2‐sided, and a *p* value <0.05 was considered statistically significant. Statistical analyses were performed using SPSS version 25.0 software (IBM Corporation) and R, version 4.0.5 (R Foundation).

## 3 RESULTS

### 3.1 Patients and baseline characteristics

In the SEER cohort, a total of 11,713 stage III cases and 6198 stage IV cases were included. Among them, there were 1448 stage IV cases receiving metastasectomy. The median follow‐up time for the whole SEER cohort and subset receiving curative‐intent resection was 39 (interquartile range [IQR], 46 months) and 47 (IQR, 49 months) months, respectively.

Among the 1135 patients in the SYSU cohort, 1011 (89.1%) patients had stage III diseases, and 124 (10.9%) patients had stage IV diseases. In the stage III patients, 755 (74.7%) patients were N1, and 256 (25.3) patients were N2. The median follow‐up time was 49 months (IQR, 43 months). In the stage IV patients, there were 66 cases with liver metastasis, 4 cases with lung metastasis, and 54 cases with other sites of metastasis, including ovarian, adrenal gland, and distant lymph nodes. All patients received curative resection of the primary tumor. For the treatment of metastatic lesions, 104 cases received resection of metastasis, 6 cases were treated with resection and ablation, and 14 patients were treated with ablation alone. The demographic and baseline clinicopathological characteristics were summarized in Table 1.

**TABLE 1:** Demographic and pathological characteristics in patient cohorts

Characteristic	SYSU cohort^a^		SEER cohort^b^		
	III (*N* = 1011)	IV (*N* = 124)	III (*N* = 11713)	Curative‐IV (*N* = 1448)	IV (*N* = 6198)
Age, years, Median (IQR)	60 (49–68)	57 (46–66)	68 (57–78)	61 (51–71)	64 (54–75)
Gender					
Male	581 (57.4)	71 (57.2)	5675 (48.4)	665 (45.9)	3158 (51.0)
Female	430 (42.5)	53 (42.7)	6038 (51.5)	783 (54.0)	3040 (49.1)
pT stage					
pT1	15 (1.5)	3 (2.41)	602 (5.13)	29 (2.00)	743 (12.0)
pT2	52 (5.14)	2 (1.61)	1014 (8.7)	36 (2.5)	138 (2.2)
pT3	793 (78.4)	83 (66.9)	7251 (61.9)	646 (44.6)	2526 (40.8)
pT4	151 (14.9)	36 (29.0)	2846 (24.2)	737 (50.8)	2791 (45.1)
pN stage					
pN1	755 (74.6)		7948 (67.8)		
pN2	256 (25.3)		3765 (32.1)		
pTNM stage					
IIIA	57 (5.6)		1403 (11.9)		
IIIB	811 (80.2)		7655 (65.3)		
IIIC	143 (14.1)		2655 (22.6)		
IVA		109 (87.9)		817 (56.4)	3357 (54.2)
IVB		15 (12.0)		631 (43.5)	2841 (45.9)
Tumor location					
Left	600 (59.3)	79 (63.7)	4229 (36.1)	549 (37.9)	2342 (37.8)
Right	291 (28.7)	25 (20.1)	6170 (52.6)	751 (51.8)	3072 (49.6)
Unknown	120 (11.8)	20 (16.1)	1314 (11.2)	148 (10.2)	784 (12.7)
Differentiation					
G1–G2	935 (92.4)	119 (95.9)	8208 (70.0)	918 (63.3)	3592 (58.0)
G3–G4	76 (7.51)	5 (4.0)	3244 (27.6)	447 (30.8)	1766 (28.5)
Unknown			261 (2.22)	83 (5.73)	840 (13.6)
Histology					
Adenocarcinoma	915 (90.5)	104 (83.8)	10,006 (85.4)	1134 (78.3)	5137 (82.9)
Mucinous adenocarcinoma or signet ring cell carcinoma	96 (9.5)	20 (16.1)	1205 (10.3)	263 (18.2)	775 (12.5)
Others			502 (4.3)	51 (3.5)	286 (4.6)

Values in parentheses are percentages.

Sun Yat‐sen University.

Surveillance, Epidemiology, and End Results.

### 3.2 Advanced stage III presented poor survival outcome comparable with stage IV disease

**FIGURE 2:**
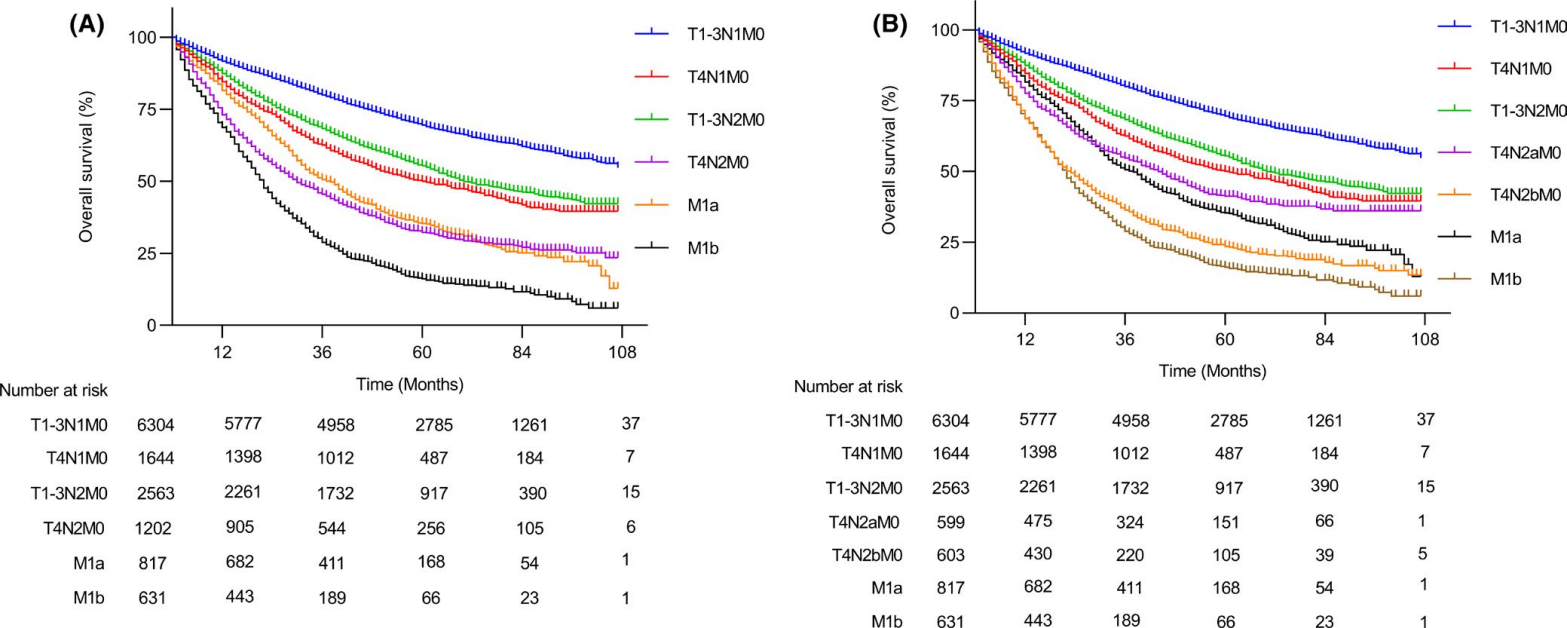
Overall survival outcome of the subset of stages III and IV colon cancer patients receiving curative‐intent resection in SEER cohort. Overall survival of patients with different TNM stages (A) and substages that were further subcategorized by N status (B)

In the SEER cohort, T4N2b patients had the worst survival outcome among stage III patients, as expected (Figure S1). In addition, T4N2b patients had similar survival outcomes with M1a patients (5‐year OS; 23.5% vs. 20.9%; *p* = 0.27). Next, to adjust the impact of survival heterogeneity introduced from the tumor resection of stage IV disease, we compared the stage III patients with the subset of stage IV patients receiving curative‐intent resection of primary and metastatic lesions in the SEER cohort. We found that the T4N2 group had a similar survival outcome compared with resected M1a (5‐year OS; 32.2% vs. 35.3%; hazard ratio [HR], 1.11; 95% CI 0.99–1.23; *p* = 0.07) (Figure 2A). Furthermore, when the N stage was further subcategorized, the T4N2b group had significantly worse survival than the resected M1a group (5‐year OS; 23.5% vs. 35.3%; HR, 1.46; 95% CI 1.28–1.66; *p* < 0.001) (Figure 2B). After the baseline variables were controlled, the adjusted Kaplan‐Meier curves suggested that the T4N2b subgroup still had a worse survival outcome (Figure S2).

### 3.3 Validation of survival outcomes of advanced stage III disease in SYSU cohort

Given the finding that the advanced group of stage III patients had worse survival outcomes compared with resected M1a group in the SEER cohort, we sought to validate it in our in‐house SYSU cohort with stage III and IV diseases. We analyzed the survival outcomes of stage III patients with different N stages and compared them with stage IV patients, and a similar result was observed.

**FIGURE 3:**
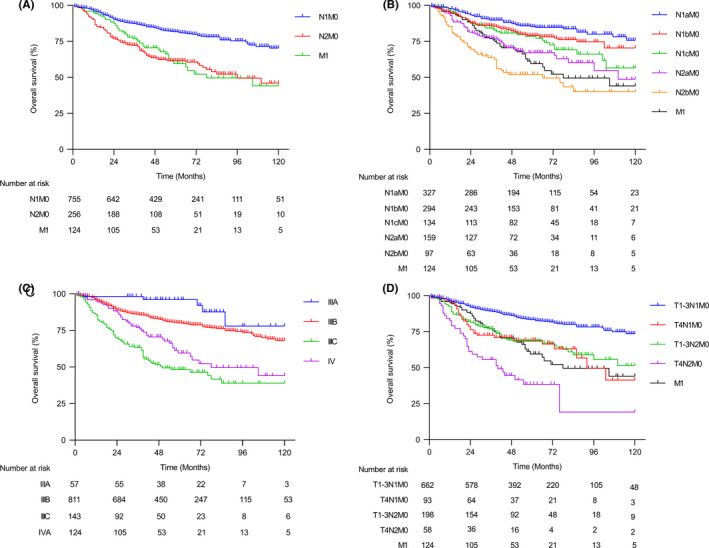
Overall survival outcome of stages III and IV colon cancer patients in SYSU cohort. Overall survival of patients with different AJCC N stages (A), N substages (B), TNM stages (C), and TNM substages (D) in the stages III–IV patients of SYSU cohort

The N2 group had similar 5‐year OS compared with the M1 group (61.6% vs. 59.5%, HR, 1.10; 95% CI 0.78–1.56; *p* = 0.58) (Figure 3A). Next, when the N stage was further subcategorized, we found a significant difference in survival outcome between N2b and other N subcategories (Figure 3B), and the N2b group had a marginal worse trend of OS compared with the M1 group (52.1% vs. 59.5%; HR, 1.54; 95% CI 1.01–2.34; *p* = 0.04). The 5‐year OS probabilities for stage IIIA, IIIB, IIIC, and IV were 96.1% (95% CI 93.4%–98.8%), 80.1% (95% CI 78.6%–81.6%), 48.2% (95% CI 43.6%–52.8%), and 59.5% (95% CI 54.2%–64.8%), respectively (Figure 3C), and the log‐rank test showed that the OS of IIIC colon cancer was significantly worse than that of stage IV (HR, 1.58; 95% CI 1.095–2.284; *p* = 0.015). Consistent with the findings in the SEER cohort, the T4N2 group represented the group with the worst survival outcomes (OS vs. M1, 38.4% vs. 59.5%; HR, 2.44; 95% CI 1.46–4.09; *p* < 0.001) (Figure 3D), and these findings was confirmed by adjusted survival analysis (Figure S3). Of note, a rapid drop of OS rates in the advanced group of stage III disease during the preceding years after surgery could be found in the both SEER and SYSU cohorts, and an intersection between the survival curves of stage III and IV patients could also be observed, indicating that the advanced group of stage III disease had worse survival outcome specifically during the years immediately after surgery (Figures 2 and 3A).

### 3.4 Multivariate analysis identified a subgroup of T4N2b patients with a high risk of death

**FIGURE 4:**
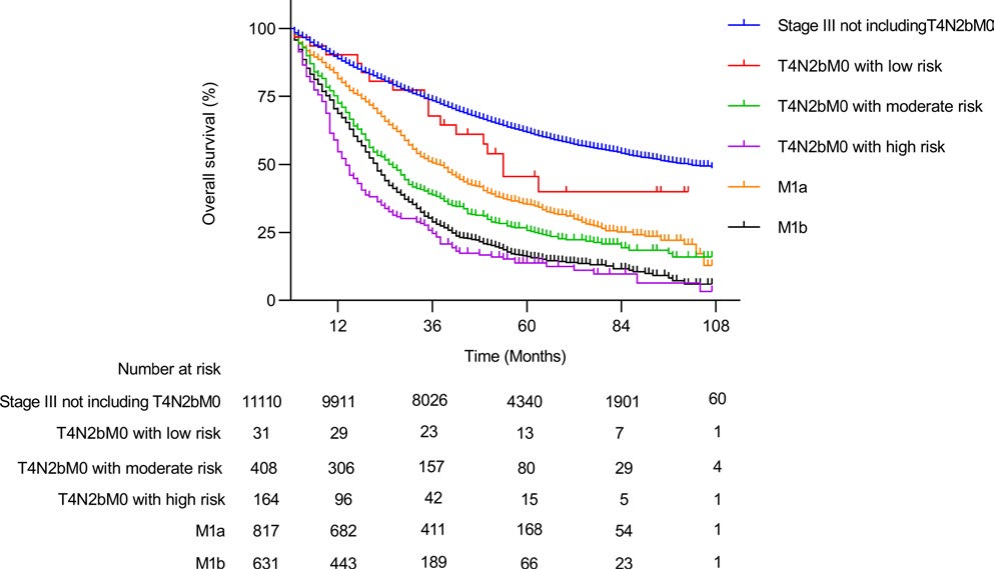
Overall survival outcome of risk groups in T4N2b patients based on the multivariate model. The T4N2b patients were divided into three groups according to the count of independent risk factors each individual patient had, including low (0 risk factors), moderate (1–2 risk factors), and high‐risk (3 risk factors) groups. The independent risk factors in the multivariate model for overall survival included right‐side tumor, poor‐undifferentiated tumor, and age over 60 years

As the T4N2b group consisted of the patients with distinct characteristics, we performed univariate and multivariate to explore the risk factors associated with OS in T4N2b patients in the SEER cohort. In the univariate analysis, the right‐side tumor, poor to undifferentiated tumor, mucinous adenocarcinoma or signet ring cell carcinoma, and old age were significantly associated with poor OS (Table S1). In the multivariate analysis, the right‐side tumor, poor‐undifferentiated tumor, and old age were identified as independent risk factors associated with poor OS (Table 2). Next, we divided the T4N2b patients into three groups according to the number of risk factors the individual patient had, including low (0 risk factors), moderate (1–2 risk factors), and high‐risk (3 risk factors) group. As shown in Figure 4, the high‐risk group had a 5‐year OS rate of only 13.8%, which was even worse than that of M1b patients (16.2%; HR, 1.24; 95% CI 1.02–1.52; *p* = 0.03). Surprisingly, the moderate‐risk group also had a significantly worse survival outcome compared with that of M1a patients (HR, 1.35; 95% CI, *p* < 0.001). After the survival analysis was adjusted by weighting the baseline variables, similar results were found (Figure S4). Together, we identified the high‐risk T4N2b patients as a subgroup that had the worst survival among colon cancer patients.

**TABLE 2:** Multivariate analysis for overall survival in the T4N2b subset receiving curative‐intent resection in the SEER cohort

Variables	Overall survival		
	HR	95% CI	*p*
Age			
<60	1		<0.001
≥60	1.49	1.20–1.84	
Tumor location			
Left‐sided	1		0.037
Right‐sided	1.26	1.02–1.57	
Histology			
Adenocarcinoma	1		0.16
Mucinous adenocarcinoma or signet ring cell carcinoma	1.17	0.94–1.46	
Grade			
Well‐moderate	1		<0.001
Poor‐undifferentiated	1.47	1.21–1.78	

## 4 DISCUSSION

This study compared stage III and IV colon cancer patients who underwent curative‐intent treatment in the SEER and SYSU cohorts, respectively. We found that the T4N2b substage showed a comparable survival outcome with whole M1a patients in the SEER cohort. Moreover, an advanced subgroup of stage III patients defined by tumors with more metastasis lymph nodes and deeper tumor infiltration showed a worse OS compared with stage IV patients who received metastasectomy. Based on the multivariate Cox model, including the right‐side tumor, poor‐undifferentiated tumor, and old age, we further identified a high‐risk group of T4N2b patients with significantly worse survival than M1b. These findings were further validated in our in‐house SYSU cohort. We further performed an adjusted survival analysis by weighting age and gender, and the results remained unchanged. Consistent with previous findings, these results further emphasize the heterogeneity within the stage III colon cancer, and current cancer care modalities may not be sufficient to achieve satisfactory survival for the high‐risk substages in stage III colon cancer.

It has been well studied that lymph nodes metastasis is a crucial prognostic factor for colon cancer, and the increasing number of metastatic nodes yields a worse survival.^12^, ^24^ The major difference between stage III and stage II colon cancer is whether the tumor has metastasized to the lymph nodes. However, according to the AJCC classification system, patients with metastasis to more than seven locoregional nodes were all categorized as N2b, which might introduce heterogeneities into N2b categories. Some recent studies had shown that the LNR, defined as the ratio of metastatic nodes relative to the number of nodes examined, served as a better tool to characterize the status of lymph nodes.^25^, ^26^ Nonetheless, the analysis based on the SEER database suggested that the LNR did not adequately represent the variation in node status, as the chance of finding a lymph node metastasis is dependent to some degree on the number of examined lymph nodes, which caused overestimation of the power of LNR and heterogeneity within similar LNR categories.^27^ In the current study, by generating a multivariate model to stratify T4N2b stage further, we identified a high‐risk subgroup of T4N2b patients that were characterized by right‐side tumor, poor‐undifferentiated tumor, and old age. This model would improve the current classification system to better guide disease management modalities for the advanced group of stage III patients.

The adverse prognosis observed in the advanced group of stage III patients raised the concern of the current management in non‐metastasis colon cancer. According to the current NCCN guideline, more intensive systemic therapies, including chemotherapy, targeted therapy, and immune checkpoint inhibitors, are recommended for some stage IV patients that were optimal with specific clinical and molecular features.^11^ The unexpectedly worse prognosis of some stage III patients compared with stage IV patients implicated that the current therapeutic modalities might be insufficient for these patients, and a classification system needs to be well established to provide intensive systemic treatment for the high‐risk stage III colon cancer selected by clinicopathological and molecular factors.

Recently, the phase II NICHE study, which intended to extend the application of immune checkpoint inhibitors as a neoadjuvant regimen for stage III colon cancer, published its interim report that the MSI‐H/dMMR group had an unprecedented rate of pathological responses (20/20, 100%).^28^ Although the trial is ongoing without long‐term results, the available data has shown possibilities of survival benefit by the application of immunotherapy in the advanced group of stage III patients with selective biomarkers.

The targeted therapy agents, including bevacizumab, cetuximab, and panitumumab, are currently recommended in patients with metastatic colon cancer^8^ and were proved to improve the progress‐free survival and OS.^29^, ^30^ There have been many randomized controlled trials (RCT) extending their application in the adjuvant therapy of non‐metastatic colon cancer,^31^, ^32^, ^33^, ^34^ but none has achieved satisfactory outcomes. However, most of these studies included the whole stage II and III patients that vary significantly in tumor nature and prognosis. Interestingly, the PETACC‐8 trial, testing FOLFOX4 and cetuximab in stage III patients, found an improved outcome, specifically in patients with pT4/N2 tumors.^34^ This finding supports the speculation of insufficient treatment from the results of our study that the patients with T4N2 colon cancer had the worst outcomes even compared with stage IV patients. Although the final follow‐up result is of insignificant difference, the NASPB C‐08 and AVANT trials, which tried to advocate bevacizumab use in the adjuvant therapy for stage II and III colon cancers, both found a significant survival benefit from bevacizumab in the first 1.5 years. Our results could support the finding that the advanced group of stage III disease had worse survival outcomes, specifically during the years before the survival curve was intersected with that of stage IV diseases.

The explanation for the findings from previous trials and the current study is uncertain, while it might be attributed to the already‐present undetectable metastasis and the effect of targeted therapies.^33^ Therefore, we anticipate that targeted agents might provide satisfactory survival benefits after careful selection of stage III patients such as those with T4N2b diseases. Considering that the addition of targeted agents seemed not to bring additional clinically relevant toxicities except for diarrhea and nausea,^33^, ^34^ future studies investigating the application of targeted agents in certain subgroups of stage III patients (e.g., those with T4N2 diseases, specific immune contexture,^35^ or sensitive molecular subtypes^36^) may draw a favorable conclusion.

The surveillance for local and distant recurrences after treatment at an early stage is one of the critical issues in the management of colon cancer. However, the underlying debate of cost‐effectiveness has lasted for decades.^37^, ^38^ According to the results of the FACS trial, the intensive surveillance schemes resulted in a higher curable rate with an odds ratio of around three compared with that of minimal surveillance.^39^ The update of its results further revealed that at a median follow‐up of 8.7 years, colon cancer could benefit from the intensive scheme.^40^ Moreover, in patients with left‐sided tumors, which has been associated with reduced risk of death in current and previous studies,^41^, ^42^ an intensive scheme could bring more survival benefits. Currently, the surveillance scheme for computed tomography (CT) scans is recommended every 6–12 months for 5 years after surgery by NCCN guidelines for all stage III colon cancer.^11^ However, a more intensive CT scans scheme is recommended for stage IV patients undergoing curative treatment every 3–6 months in the first 2 years and every 6–12 months for the next 3 years. The recommendation is based on the higher risk of stage IV and the consensus that the early resection of recurrent lesions undetectable in previous CT scans could improve survival outcomes.^43^, ^44^ The intensive surveillance scheme enables earlier detection of relapse for possible curative treatment.^45^ The current study demonstrated that the advanced group of stage III patients had worse survival outcomes than stage IV patients receiving curative treatment. Considering the high risk of this subgroup, it is reasonable to refer to the surveillance scheme of stage IV diseases and provide additional and intensive surveillance for these high‐risk stage III patients, which is promising to improve their survival outcomes in the future well‐designed RCTs.

This study had some limitations. First, this study was limited by its retrospective design. In addition, to reach the comparability between stage III and stage IV patients, resected stage IV patients were selected for analysis in the SYSU cohort, which might introduce selection bias. The sample size of SYSU cohort limited the further subgroups analysis, though the difference was significant with sufficient statistical power in the overall analysis. Finally, we could not conclude the benefits of more intensive treatment and surveillance modalities for advanced stage III patients based on the current observative study, which needs investigation in the upcoming clinical trials in multiple centers for further validation.

In conclusion, by comparing stage III and stage IV colon cancer patients who underwent curative‐intent treatment in two integrated cohorts, we found that stage T4N2b is a high‐risk group of stage III patients with a comparable or worse survival outcome compared with resectable stage IV patients. This finding raised the concern that the current therapeutic modalities might be insufficient for this high‐risk group. Additional targeted therapy, immunotherapy, and intensive surveillance might be a surrogate option in future clinical trials to achieve better survival outcomes.

## ETHICS APPROVAL

Before conducting the study, we obtained a review board approval from the Sixth Affiliated Hospital of Sun Yat‐sen University.

## CONFLICT OF INTEREST

The authors declare no conflict of interest.

## Supporting information

Supplementary Material

## Data Availability

The data from the SYSU cohort that support the findings of this study are available on request from the corresponding author. The data are not publicly available due to privacy or ethical restrictions. The data that support the findings of this study are openly available in the Surveillance, Epidemiology, and End Results database.
