# Supplementary material for: Current treatment and surveillance modalities are not sufficient for advanced stage III colon cancer: Result from a multicenter cohort analysis
Source: Cancer Med. 2021 Nov 16;10(24):8924–33. doi: 10.1002/cam4.4417 (PMC8683545; doi:10.1002/cam4.4417)
Supplement: Supplementary file 1 — Supplementary Material [file CAM4-10-8924-s001.docx]

Supplements

**1. sTable 1** Univariate Analysis for Overall Survival in T4N2b Patients from the SEER Curative-Intent Cohort

**2. Figure S1** Overall survival of patients with different AJCC TNM substages in the whole SEER cohort

**3.** **Figure S2** Adjusted overall survival outcome of the subset of stage III and IV colon cancer patients receiving curative-intent resection in SEER Cohort

**4.** **Figure S3** Adjusted overall survival outcome of stage III and IV colon cancer in SYSU cohort

**5.** **Figure S4** Adjusted overall survival outcome of different risk groups in T4N2b patients based on the multivariate model

| **sTable 1. Univariate Analysis for Overall Survival in T4N2b Patients from the SEER Curative-Intent Cohort** | | | |
| --- | --- | --- | --- |
| **Variables** | **OS** | | |
|  | **HR** | **95% CI** | ***P*** |
| Sex |  |  | 0.11 |
| Female | 1 |  |  |
| Male | 0.86 | 0.72-1.03 |  |
| Age |  |  | <0.001 |
| <60 | 1 |  |  |
| ≥60 | 1.53 | 1.24-1.88 |  |
| Race |  |  | 0.99 |
| White | 1 |  |  |
| Black | 1.00 | 0.71-1.40 |  |
| Site |  |  | 0.001 |
| Left | 1 |  |  |
| Right | 1.44 | 1.16-1.79 |  |
| Grade |  |  | <0.001 |
| Well-Moderate | 1 |  |  |
| Poor-Undifferentiated | 1.54 | 1.27-1.86 |  |
| Histology |  |  | 0.04 |
| Adenocarcinoma | 1 |  |  |
| Mucinous adenocarcinoma or signet ring cell carcinoma | 1.26 | 1.00-1.56 |  |


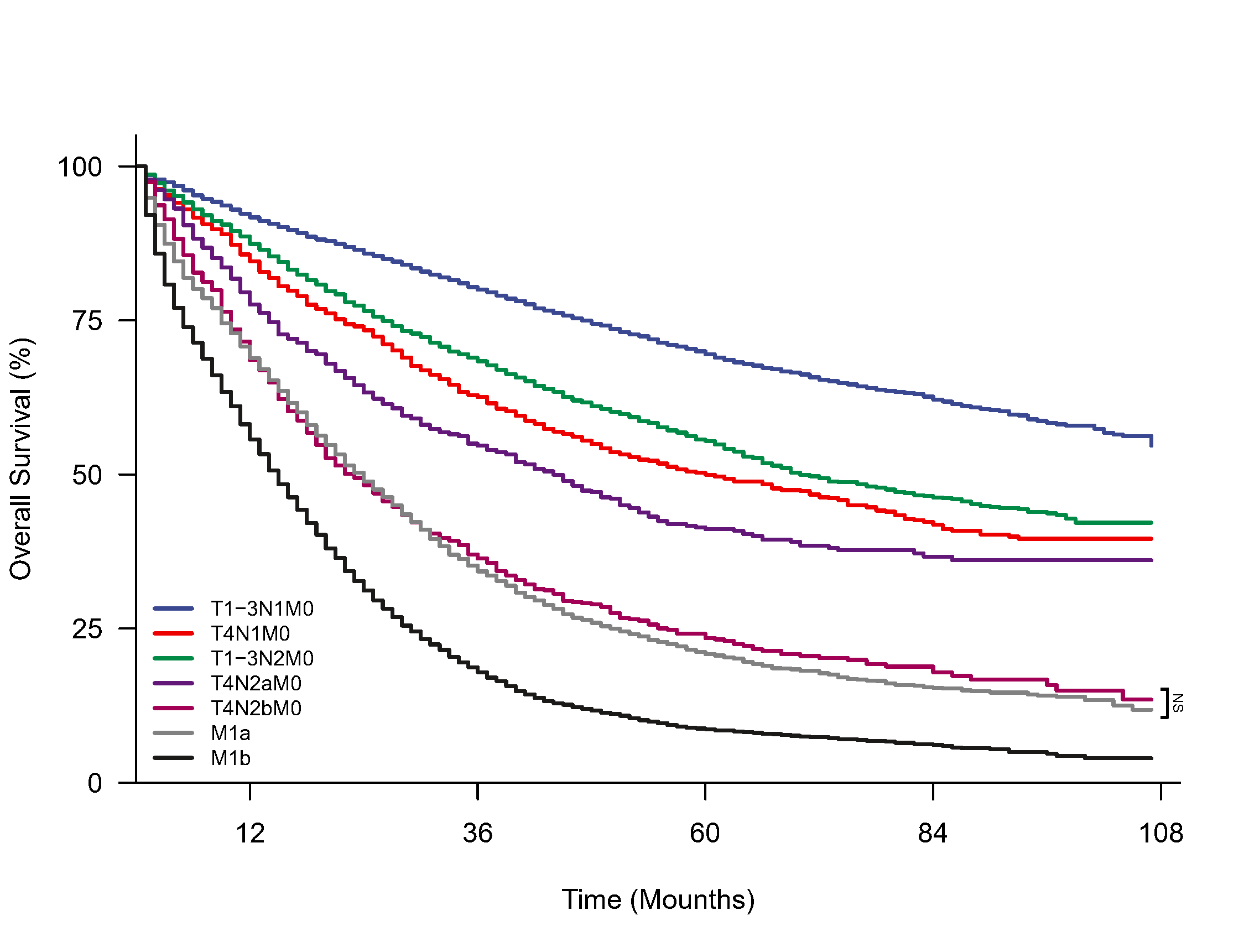


**Figure S1** Overall survival of patients with different AJCC TNM substages in the whole SEER cohort


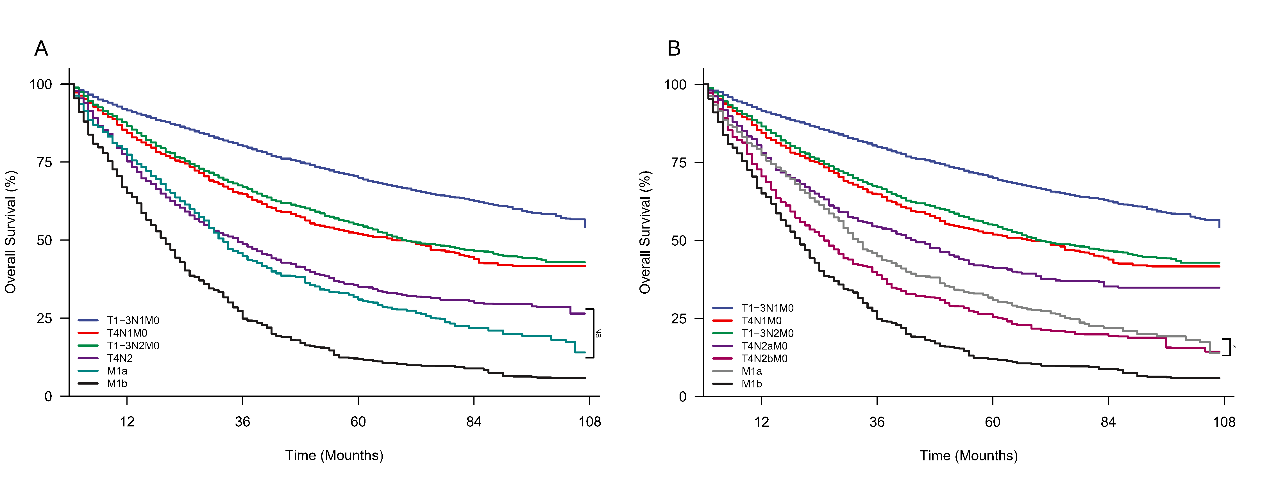


**Figure S2** Age and sex-adjusted overall survival outcome of the subset of stage III and IV colon cancer patients receiving curative-intent resection in SEER Cohort

Adjusted overall survival of different AJCC TNM substages (A), divided T4N2b patients by subcategories of N status (B).


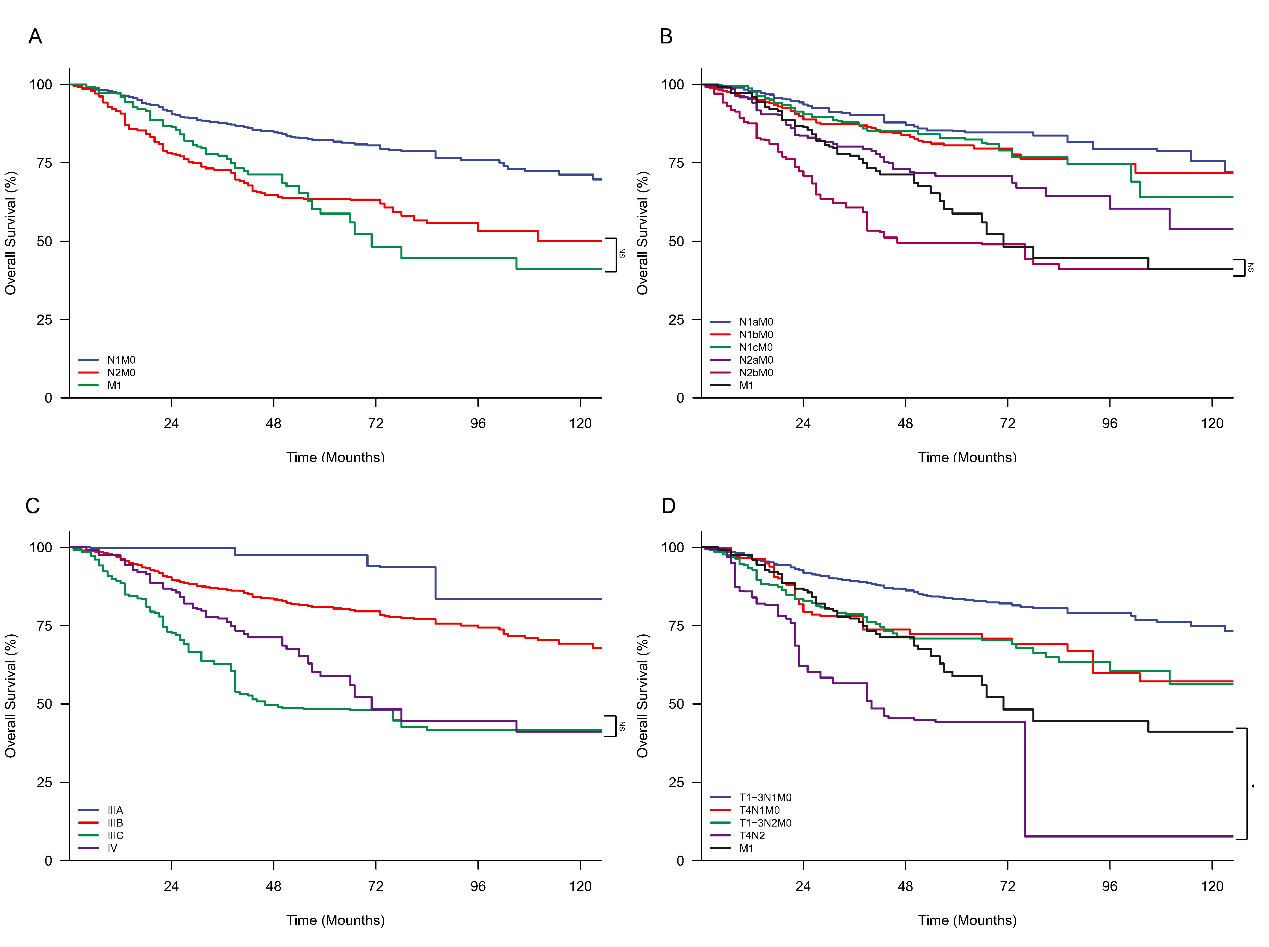


**Figure S3** Age and sex-adjusted overall survival outcome of stage III and IV colon cancer in SYSU cohort

Adjusted overall survival of different AJCC N stages (A), N substages (B), TNM stages (C), and TNM substages (D) in the stage III-IV patients of SYSU cohort.


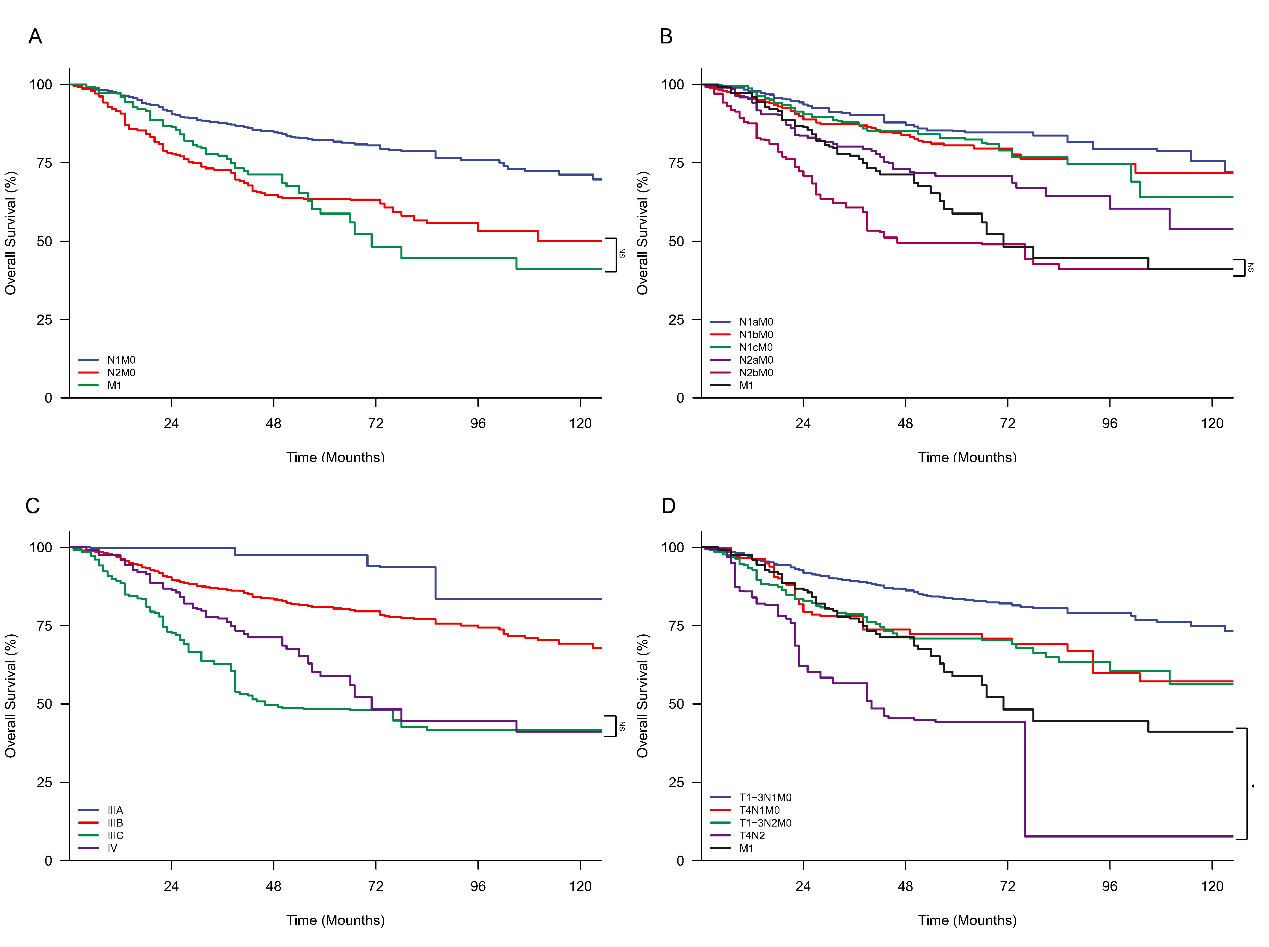


**Figure S4** Age and sex-adjusted overall survival outcome of different risk groups in T4N2b patients based on the multivariate model

The T4N2b patients were divided into 3 groups according to the number of independent risk factors the individual patient had, including low (0 risk factors), moderate (1-2 risk factors), and high-risk (3 risk factors) group. The independent risk factors in the multivariate model for overall survival included right-side tumor, poor-undifferentiated tumor, and age over 60 years.
